# Inflammation—The new treatment target for ischaemic stroke prevention

**DOI:** 10.3389/fstro.2023.1241506

**Published:** 2023-10-23

**Authors:** Sarah Gorey, John J. McCabe, Peter J. Kelly

**Affiliations:** ^1^Health Research Board (HRB) Stroke Clinical Trials Network Ireland (SCTNI), Dublin, Ireland; ^2^School of Medicine, University College Dublin, Dublin, Ireland; ^3^Stroke Service, Mater Misericordiae University Hospital, Dublin, Ireland

**Keywords:** ischaemic stroke, atherosclerosis, large artery atherosclerosis (LAA), inflammation, high sensitivity C-reactive protein (hsCRP), interleukin-6 (IL-6)

## Abstract

Recurrent vascular events after stroke are common despite contemporary therapies and there is an unmet clinical need for improved secondary prevention. Inflammation is a probable causal factor in first and recurrent stroke and is a promising therapeutic target. Blood biomarkers of inflammation may also improve risk stratification and patient selection for intensive prevention therapies. We review the pathogenic role of inflammation in stroke and atherosclerosis, examining data from observational and genetic studies as well as randomized controlled trials of anti-inflammatory agents in stroke and cardiac disease. We discuss the potential applications for inflammatory biomarkers in stroke care and evaluate some of the uncertainties and controversies in this field.

## Introduction

According to the Global Burden of Diseases, stroke is the third-leading cause of death and disability combined (GBD 2019 Stroke Collaborators, 2019). Although stroke incidence is falling in high-income countries, globally stroke prevalence is rising (Koton et al., 2014; GBD 2019 Stroke Collaborators, 2019). The rise in absolute number of incident strokes is driven by increases among people younger than 70 years and in lower-income countries (GBD 2019 Stroke Collaborators, 2019). The risk of stroke recurrence is high, between 20 and 60% at 5 years in registries and population studies (Mohan et al., 2011; Boulanger et al., 2018; Skajaa et al., 2022). Prevention of recurrent stroke is a global public health priority (Norrving et al., 2018; Kleindorfer et al., 2021). Inflammation is an independent contributor to first and recurrent stroke risk (Ridker, 2017). Accumulating evidence points to the role of inflammation in the pathogenesis of atherosclerosis and other stroke etiologies, and its potential as a prognostic indicator and therapeutic target for stroke prevention (Pearson et al., 2003; Kelly et al., 2021b).

In this review we outline the evidence supporting the role of inflammation in atherosclerotic disease and stroke pathogenesis ranging from laboratory to imaging studies, genetic epidemiology studies and randomized controlled trials. We will also discuss controversies and uncertainties, outline knowledge gaps and potential for clinical translation.

## Inflammation and pathogenesis of atherosclerosis

Experimental and clinical data indicate that atherosclerosis is a chronic maladaptive inflammatory disorder associated with intimal accumulation of modified lipids. Inflammation is pivotal in the development, progression, and rupture of atherosclerotic plaque, leading to thrombo-embolic events including stroke (Libby et al., 2019). We will describe key inflammatory processes in endothelial adhesion of monocytes, recruitment of macrophages and their conversion to foam cells, activation of the NLRP3 inflammasome and down-stream release of pro-inflammatory cytokines, as well as in the dynamic process driving atherosclerotic stability or rupture.

**Figure 1:**
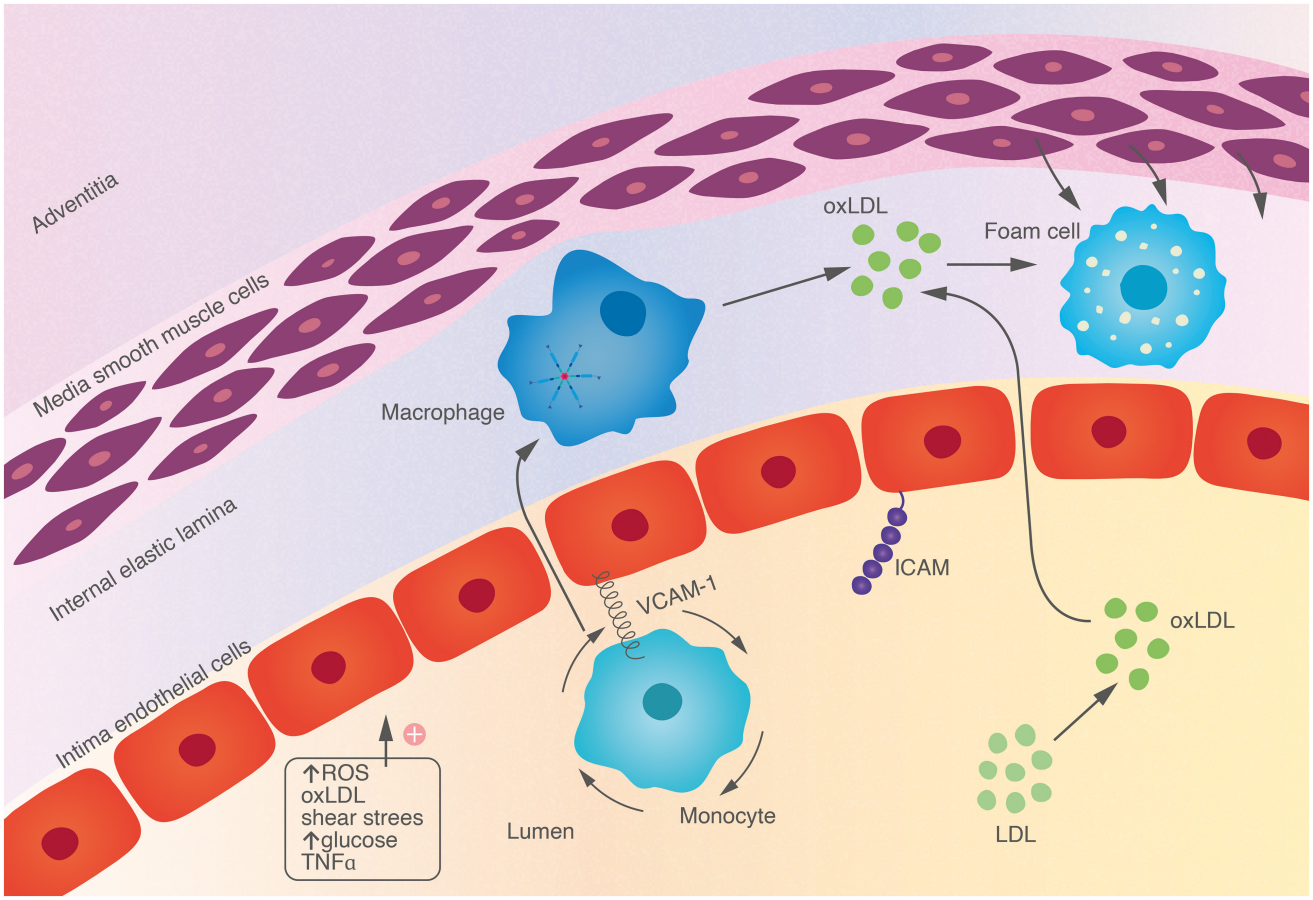
Endothelial cells are activated by a variety of endogenous and environmental stressors, leading to expression of adhesion molecules on the surface. This facilitates adhesion and rolling of monocytes which migrate into tissue, engulfing oxidized lipid particles, becoming foam cells, a hallmark of early atherosclerosis.

Endothelial stimulation, by proinflammatory cytokines or irritative stimuli (smoking, hypertension, hypercholesterolaemia) leads to expression of adhesion modules [e.g. vascular cell adhesion molecule 1 (VCAM-1) and intercellular adhesion molecule 1 (ICAM-1)] which facilitate adherence and rolling of monocytes and lymphocytes along the endothelium. Chemokines, such as interleukin-1 (IL-1), tumor necrosis factor (TNF) and monocyte chemoattractant protein-1 (MCP-1), direct migration of these leukocytes into the arterial intima (Figure 1). Impaired barrier function of damaged endothelium facilitates entry of LDL particles. Mononuclear phagocytes in the circulation differentiate into macrophages within the intima which phagocytose lipid particles becoming foam cells, an early hallmark of atherosclerosis. Cholesterol crystals form within macrophages, further dysregulating intracytoplasmic vesicular membranes, triggering activation of the NLRP3 (nucleotide binding oligomerisation domain like [NOD-like] receptor, pyrin domain containing protein 3) inflammasome, and stimulating the complement system (Baumer et al., 2021). The adaptive immune system also contributes to atherogenesis with T-helper cells and their associated cytokines interferon-gamma (IFNγ), interleukin-12 and interleukin-18 acting in concert with innate immune cells in the intima to stimulate proinflammatory cytokines.

Clonal haematopoiesis of indeterminate potential (CHIP) has recently been implicated in atherosclerosis and is associated with stroke and cardiovascular disease (Jaiswal et al., 2017). CHIP occurs in association with somatic mutations associated with increased risk of leukemia. It is associated with mutations in genes, for example TET2, which alter DNA methylation, altering expression of inflammatory cytokines IL-1 and interleukin-6 (IL-6) (Libby et al., 2019).

Vascular smooth muscle cells undergo “phenotypic switching” in response to circulating inflammatory factors, promoting migration to the intima, and acquisition of macrophage-like features (phagocytosis of lipid), and fibroblast-like features (generation of extracellular matrix components, which may contribute to stabilizing plaque) (Bennett et al., 2016).

## Inflammation, plaque progression, and rupture

Atherosclerotic plaques progress through continued accumulation of lipid, macrophage-phagocytosis and ineffective effocytosis, leading to formation of a necrotic core with a collagen-rich fibrous cap. Macrophages produce collagenolytic matrix metalloproteinases (MMPs) which weaken the cap, making it prone to rupture. Interferon-gamma (IFNγ) produced from T-cells also impairs collagen synthesis (Libby et al., 2019).

Atherosclerosis develops in a dynamic and cyclical fashion in response to the changing balance of pro and anti-inflammatory signals (Libby, 2021a). Macrophages become polarized depending on inflammatory signals in their environment: circulating IFNγ and TNF produce M1, “pro-inflammatory” macrophages, which secrete pro-inflammatory cytokines, including IL-1 and IL-6. M2 “anti-inflammatory” macrophages have regulatory roles and facilitate fibrosis (Libby, 2021b). Although the spectrum of macrophage activation is wider than M1/M2 subtypes, M2 macrophages are associated with stable plaque whereas high expression of M1 macrophages is associated with plaque rupture (de Gaetano et al., 2016). Anti-inflammatory signals, such as IgM secreted by B1-lymphocytes or IL-10 released from T-helper cells, may also slow plaque progression. Plaque rupture occurs due to interplay of lipid accumulation, necrosis and accumulation of inflammatory cells and collagenases which degrade the fibrous cap of the plaque (Libby et al., 2019). Intra-cap cholesterol crystal formation is also associated with plaque rupture (Katayama, 2020).

## Inflammation and thrombosis

After plaque rupture, tissue factor (TF) release from the plaque core initiates generation of fibrin, activation of platelets and the coagulation cascade (Nording et al., 2015). Platelets have immune functions and may potentiate NLRP3 inflammasome function (Rolfes et al., 2020). Platelets also recruit leukocytes mediated by P-selectin and ICAM-1, aggregate with neutrophils and modulate neutrophil function contributing to neutrophil extracellular trap (NET) formation (Nording et al., 2015). NETs have a role in thrombus formation and are stimulated by leukocytes and IL-1β from upstream NLRP3 inflammasome activation. Separately, thrombin is formed as a result of coagulation cascade activation when TF binds to factor VII (a) (Olie et al., 2018).

## The inflammasome-IL-1-IL-6-CRP pathway

**Figure 2:**
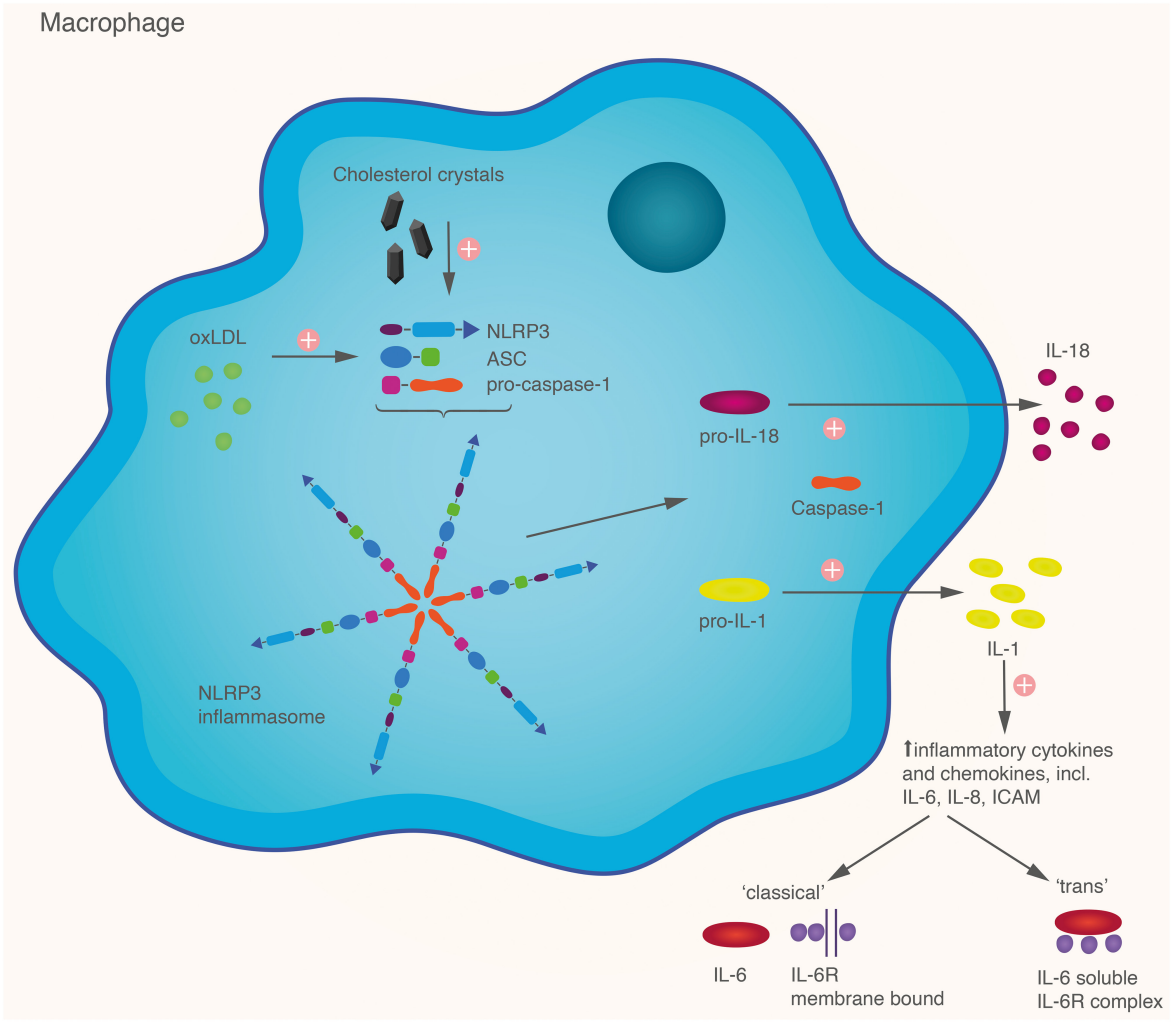
Within the macrophage, cholesterol crystals and oxidized LDL particles trigger the assembly of the NLRP3 inflammasome, comprised of subunits CARD-ASC, NLRP3 receptor and pro-caspase-1. Activation of the inflammasome generates active caspase-1 which cleaves pro-IL-1 and pro-IL-18 to active IL-1 and IL-18. These are released from the cell and propagate further inflammatory cascades. IL-1 is responsible for the activation of IL-6, which can signal in a “classical” fashion via membrane bound receptors, or via “trans-signaling” by forming a soluble complex with IL-6R, allowing IL-6 to be active in a wide range of tissues.

Within the endoplasmic reticulum of macrophages, NLRP3 (nucleotide binding oligomerisation domain like [NOD-like] receptor, pyrin domain containing protein 3) inflammasome assembly is stimulated by intracytoplasmic danger signals, including uric acid and cholesterol crystals (Martínez et al., 2018). The NLRP3 inflammasome is comprised of three components: a NLRP3 receptor (a type of Toll-like receptor), adaptor protein apoptosis associated speck-like protein containing caspase and activation recruitment domain (CARD-ASC) and the cysteine protease caspase-1. ASC acts as a functional link between caspase-1 and NLRP3 components (Figure 2). A two-step process is required for inflammasome activation: first the toll-like receptor is primed by a specific activating intracellular stimulus followed by caspase-1 mediated cleavage of pro-IL-1β and pro-IL-18 to their active forms (Duewell et al., 2010; Rajamäki et al., 2010).

IL-1β is an upstream pro-inflammatory cytokine, which further auto-stimulates IL-1β expression and promotes expression of other inflammatory cytokines such as TNF-α (Ridker, 2016). IL-1β stimulates the production of intra-plaque and systemic interleukin-6 (IL-6) from inflammatory cells, endothelium, smooth muscle cells and adipocytes (Libby and Rocha, 2018). IL-6 signaling occurs through two distinct pathways: ‘classical signaling' (binding of the IL-6 molecule to its membrane-bound IL-6 receptor, expressed on selected tissue types such as hepatocytes and immune cells, mainly macrophages and T-cells) and ‘trans-signaling' (whereby IL-6 binds to soluble IL-6-R to form circulating protein-receptor complexes, which have the capacity to bind to a wide range of tissue cell membranes via the ubiquitously-expressed gp130 subunit) (Del Giudice and Gangestad, 2018; Libby and Rocha, 2018). “Downstream” stimulation of hepatocytes by IL-6 increases expression of circulating inflammatory protein markers such as fibrinogen, plasminogen activator inhibitor-1, serum amyloid-A and C-reactive protein (CRP).

Accumulating evidence indicates that the inflammasome-IL1-IL6-CRP axis is central in pathophysiology of atherosclerosis, thrombo-embolic events, and stroke. In addition to biomarker and pathological studies (below), a genetic epidemiological (Mendelian randomization) study of genetic proxies for lower IL-6 activity demonstrated that reduced IL-6 activity was associated with a reduced risk of ischaemic stroke. The odds ratio [OR] was 0.89, 95% CI 0.82–0.97, with an even stronger association for large artery atherosclerosis (LAA) and small vessel stroke (SVD) stroke subtypes (LAA: OR 0.76, 95% CI0.62–0.93 and SVD: OR 0.71, 95% CI 0.59–0.86) but not for cardioembolic stroke (Georgakis et al., 2020). Because of the low likelihood of confounding inherent in the genetic epidemiological design, these findings suggest IL-6 has a directly causal role in stroke pathogenesis. Further support of a causal relationship derives from the CANTOS randomized controlled trial (RCT), which demonstrated that canakinumab, a selective IL-1β monoclonal antibody, reduced the risk of cardiovascular events in patients with coronary disease, with greatest benefit in patients with on-treatment CRP < 2mg/L and/or IL-6 below the median value of 1.65 ng/L (Ridker et al., 2017, 2018a,b).

## CRP and IL-6 as stroke risk biomarkers

### CRP

CRP and IL-6 may have clinical utility as markers of risk or response to treatment. CRP is a sensitive acute phase reactant and non-specific marker of inflammation (Sproston and Ashworth, 2018). CRP is stable in blood and easily measurable with widely available standardized assays. In an individual participant meta-analysis of 160,309 people free of cardiovascular disease or stroke, log-transformed CRP concentration had a linear association with conventional cardiovascular risk factors, and a log-linear relationship with first ischaemic stroke (Emerging Risk Factors Collaboration et al., 2010). The risk ratio for ischaemic stroke per 1-standard deviation (SD) increment in log_e_CRP (equivalent to a three-fold increase on the natural scale) was 1.27 (95% CI 1.15–1.68) after adjustment for established vascular risk factors. However, CRP was not specific for stroke and was also associated with deaths from cancer, lung disease and non-vascular causes (Emerging Risk Factors Collaboration et al., 2010). Consistent with this observation, genome wide association studies have suggested that CRP is unlikely to be causal in atherogenesis (Dehghan et al., 2011).

Nevertheless, CRP, due to its stability and ease of measurement, remains an attractive biomarker to stratify risk of recurrent stroke or vascular events including fatal and non-fatal post-stroke myocardial infarction. A case-control study of 680 patients with minor stroke or TIA, with systematic exclusion of patients with confounding infection or pro-inflammatory diseases, found that higher baseline levels of IL-6, IL-8 and high sensitivity (hs) CRP independently predicted one-year recurrent vascular events (Coveney et al., 2021). An individual participant data meta-analysis of 8,420 participants after stroke, found that serum hsCRP was associated with increased risk of recurrent stroke and major cardiovascular events (MACE) after adjustment for vascular risk factors and treatment (recurrent stroke RR 1.12, 95% CI 1.04–1.21, and MACE RR 1.19, 95% CI 1.09–1.29, both per unit increase log_e_hsCRP) (McCabe et al., 2023). When analyzed in quarters, a dose-dependent increase in risk of MACE was evident: those with the highest hsCRP levels were one-third more likely to have a recurrent event compared to those with the lowest hsCRP levels (for MACE, Q4 vs. Q1: RR 1.33, 95% CI 1.08–1.65; for recurrent stroke, Q4 vs. Q1: RR 1.16, 95% CI 0.93–1.43). After stratification by stroke etiology, hsCRP was only associated with MACE for stroke of undetermined etiology (Q4 vs. Q1: RR 1.45, 95% CI 1.04–2.03) but not for other stroke subtypes (ESOC, 2023). In an analysis of data from the CHANCE trial comprising 3,044 participants, patients with CRP > 3 mg/L after minor stroke or TIA had a higher risk (adjusted HR 1.46, 95% CI 1.08–1.98) of 90-day recurrent stroke compared to those with CRP < 1 mg/L (Li et al., 2016).

American Heart Association guidelines now suggest statin therapy in those at intermediate risk of first vascular event if hsCRP ≥ 2 mmol/L (Arnett et al., 2019). For stroke, the CNSR-III investigators examined residual inflammatory risk (RIR, risk associated with high CRP in patients with low on-treatment LDL) in a multicentre prospective cohort of over 10,000 stroke and TIA patients in China. They reported RIR was independently associated with recurrent stroke after adjustment for vascular risk factors (HR 1.18, 95% CI 1.00–1.30), although this attenuated after further adjustment for antiplatelet and statin therapy (HR 1.31, 95% CI 0.99–1.76). This association was strongest for large artery strokes (adjusted HR 1.69, 95% CI 1.06–2.67) which is biologically consistent (Li et al., 2021).

### IL-6

Although less-widely studied, IL-6 may also have utility as a marker of risk in patients with stroke. A meta-analysis of 11 studies comprising over 27,000 stroke-free participants demonstrated that a 1-standard deviation increment in log-transformed IL-6 was associated with an increased risk of first ischaemic stroke over a decade of follow-up (RR 1.19, 95% CI 1.10–1.28). In a factorial Mendelian randomization study of RIR in 408,225 participants in the UK Biobank, patients with both sub-median IL-6 and LDL were at lowest risk of any future cardiovascular event (OR 0.92, 95% CI 0.9–0.95) while those with only one of either biomarker below the median had identical, higher risk (OR 0.96, 95% CI 0.93–0.98). However, these findings were not significant for the outcome of ischaemic stroke alone (Georgakis et al., 2022).

The BISC study of individual participant data (IPD) in 8,420 patients with prior stroke or TIA and 18,920 person-years of follow-up, demonstrated that baseline IL-6 measured after stroke or TIA was independently associated with recurrent MACE (RR 1.12, 95% CI 1.04–1.21) and recurrent stroke (RR 1.09, 95% CI 1.00–1.19) per unit increase in log_e_ IL-6, after adjustment for vascular risk factors and treatment. When the highest quarter of IL-6 levels was compared with the lowest quarter, raised IL-6 was strongly associated with both MACE and recurrent stroke (MACE RR 1.35, 95% CI 1.09–1.67; recurrent stroke RR 1.33, 95% CI 1.08–1.65). These hypothesis-generating data suggest that IL-6 may have clinical utility as a more specific inflammatory risk marker for predicting recurrent stroke and vascular events compared with CRP.

Challenges to routinely measuring IL-6 include absence of an agreed “normal” range, variability due to trauma, infection, age, and vascular co-morbidities, and sensitivity to sample processing factors such as delay to analysis and temperature (McElvaney et al., 2021). Therefore, more data and improved standardization are needed to validate its use before considering for routine measurement in stroke clinical practice.

Other challenges exist before routine blood inflammatory biomarker measurement can be translated to clinical practice for risk stratification. First, inflammatory markers are non- specific and rise in response to infection or acute cerebral infarction. Interpretation of measurements taken acutely must consider infarct size and etiology, the timing of phlebotomy and the analytic methods used. Second, standardized thresholds denoting “high-risk” are not yet validated in stroke patients. Third, the added value of inflammatory markers to established risk markers for outcome prediction and treatment decisions remains to be demonstrated.

Alternatively, combinations of inflammatory markers may be more specific for certain etiologies of stroke and for specific prognostic functions. Researchers in Zurich have developed a Biomarker Panel Index for risk stratification: they have derived a combination of 16 biomarkers (including von Willebrand-factor, interleukin-18-binding-protein, interleukin-2-receptor-subunit-alpha, CC-chemokine-ligand-15, among others) which independently predict mortality and cardioembolic stroke etiology with good accuracy (AUC mortality 0.93, 95% CI 0.89–0.96, and AUC CES 0.70, 95% CI 0.64–0.77). These results require external validation (Bicvic et al., 2022). Machine learning technologies and more widespread use of proteomic analysis may support a more individualized approach to risk assessment using inflammatory markers.

## Inflammation and stroke subtypes

### Large artery atherosclerosis

The association between inflammation and stroke is strongest and most biologically plausible for large artery stroke. When BISC data was stratified by stroke etiology, IL-6 was associated with recurrent MACE in those whose index stroke/TIA was caused by large artery atherosclerosis (LAA) (adjusted RR 2.30, 95% CI 1.21–4.36), but also for stroke of undetermined cause (adjusted RR 1.78, 95% CI 1.19-2.66) and small vessel occlusion (adjusted RR 1.71, 0.99–2.96), but not for cardioembolic stroke (highest [Q4] vs. lowest quarters [Q1] of the distribution). A similar trend was seen for the outcome of recurrent stroke: (Q4 vs. Q1, in LAA stroke: RR 2.22, 95% CI 1.14-5.33; for undetermined cause: RR 1.65, 95% CI 1.09–2.5; for small vessel stroke: RR 1.66, 95% CI 0.91–3.02) (ESOC, 2023).

Further evidence that inflammation is important in the pathogenesis of large artery stroke comes from histological studies of carotid plaques and imaging studies of carotid atherosclerosis.

Pathological studies of symptomatic carotid plaque have reported dense inflammatory infiltrate, high levels of IL-6, and acute thrombus composed of aggregated platelets and fibrin on the plaque surface (Spagnoli et al., 2004). The density of macrophages within the fibrous cap is independently associated with plaque rupture (Redgrave et al., 2008). Ruptured plaques express high concentrations of MMP-8 which is expressed by macrophages (Molloy et al., 2004). MMP-8 degrades fibrillar collagen which contributes to plaque stability. The Oxford Plaque Study (OPS) systematically performed histological study of symptomatic carotid plaques from over 500 consecutive endarterectomy patients. Dense plaque inflammation and macrophage infiltration was strongly associated with plaque rupture (OR 3.39, 95% CI 2.31–4.98). Carotid plaques which were excised at later times from the index stroke/TIA event had less unstable features, supporting the concept of continuous plaque remodeling (Redgrave et al., 2006). The AtheroExpress investigators reported that plaque harvested within 30 days of stroke or TIA expressed high levels of pro-inflammatory cytokines IL-6, IL-8, MMP-8, MMP-9 and dense macrophage infiltration. However, as time from event to plaque harvest increased, the density of macrophage staining within the plaques decreased, as did IL-6, IL-8 and MMP-8 concentrations, indicating that in atheromatous plaques, remodeling is mediated by molecular fluctuations which influence plaque stability (Peeters et al., 2009).

These findings were extended by results from a Dublin observational cohort study of patients with recent stroke caused by ipsilateral carotid stenosis, which demonstrated that histological features of plaque inflammation were independently associated with stroke recurrence. Patients who experienced early stroke recurrence before carotid endarterectomy had more dense macrophage infiltration (OPS grade ≥3 91.7% vs. 37.5% in recurrence-free, *p* = 0.002) extensive fibrous cap disruption(90.9% vs. 37%, *p* = 0.004) and more neovascularisation (OPD grade ≥2 83.3% vs. 43.8%, *p* = 0.04) in their carotid plaque samples (Marnane et al., 2014).

High resolution plaque MRI reliably identifies markers of plaque instability, including intraplaque hemorrhage, lipid-rich necrotic core or thin or ruptured fibrotic plaque, which correlate closely with histological evidence of inflammation. Injection of the macrophage labeling agent, USPIO (ultrasmall superparamagnetic particles of iron oxide) can identify plaque inflammation on MRI and USPIO accumulation in macrophages on MRI corresponds to histological macrophage density in endarterectomy samples (Trivedi et al., 2006).

^18^F^−^flurodeoxyglucose-positron emission tomography (^18^FDG-PET) is a more widely-studied non-invasive imaging tool which quantifies inflammation in carotid plaque. ^18^FDG-PET is a validated technique for imaging vascular inflammation associated with atherosclerosis, due to the affinity of metabolically-active macrophages for ^18^FDG, a glucose analog (Rudd et al., 2002). In stroke/TIA patients, carotid plaque inflammation, quantified by ^18^FDG uptake, is associated with early and late (5 year) ipsilateral stroke recurrence, independent of the degree of carotid stenosis and other risk factors (Marnane et al., 2012; Kelly et al., 2019; McCabe et al., 2021). A clinical risk prediction score, SCAIL (symptomatic carotid atheroma inflammation lumen-stenosis) has been derived and validated, which combines the severity of carotid stenosis with plaque inflammation imaged by PET and improved identification of patients with early and 5-year recurrent ipsilateral stroke compared to carotid stenosis alone (Kelly et al., 2020; McCabe et al., 2021). Imaging guidelines have standardized the practice of plaque imaging using PET. However its translation into routine clinical practice is limited by high cost, complex protocols and low availability (Bucerius et al., 2016). The advent of hybrid PET/MRI scanners will allow for comprehensive assessment of atherosclerotic plaque and more accurate non-invasive detection of high-risk features (Evans et al., 2020).

### Inflammation and lacunar stroke

Studies have described specific associations between inflammatory markers and small vessel stroke. Higher concentrations of blood inflammatory markers (TNFα, IL-6 and ICAM-1) were independently associated with neurological deterioration and poor outcome in a series of 113 patients with lacunar stroke (Castellanos et al., 2002). Similarly, nested within the SPS3 trial, the LIMITS study found that among 1,244 patients with lacunar stroke, hsCRP, TNFαR-1 and IL-6 predicted recurrent stroke and vascular events. The HR for the highest quartile of hsCRP for recurrent stroke was 2.32 (95% CI 1.15–4.68), and for MACE was 2.04 (95% CI 1.14–3.67) (adjusted for risk factors) (Elkind et al., 2014). The associations for TNFαR-1 and IL-6 did not persist after full adjustment when analyzed per quarter of the distributions. However both inflammatory markers were associated with recurrent MACE when analyzed as continuous variables (TNFαR1, adjusted HR 1.21, 95% 1.05–1.41; IL-6, adjusted HR 1.1, 95% 1.02–1.19, both per SD increase) (Boehme et al., 2016).

Similarly, in the MEGASTROKE study, higher genetically-predicted IL6R (and lower IL6 signaling) was associated with protection against first-ever small vessel stroke (OR 0.939, 95%CI 0.909–0.970) (Chen et al., 2022). CHIP has also been associated with lacunar stroke (HR 1.55, 95% CI 1.29–1.82), as well as haemorrhagic stroke (HR 1.25, 95% CI 1.01–1.51) in a biobank study of 78,752 individuals (Bhattacharya et al., 2022).

### Inflammation and cardioembolic stroke

Atrial fibrillation (AF) is associated with a pro-inflammatory state. In 4,893 patients with AF in the RE-LY trial, raised IL-6 was independently associated with a two-fold risk of stroke or systemic embolism (Q4 vs. Q1, HR 2.03, 95% CI 1.27–3.26), adjusted for vascular risk factors. This risk attenuated after further adjustment for cardiac and renal biomarkers NT-pro-BNP, troponin I and cystatin C (Aulin et al., 2015). Similarly, in a cohort of 425 patients with AF-related cardioembolic stroke (CES) from the Fukuoka Stroke Registry, CRP was independently associated with recurrent stroke at one year (HR 1.02, 95% CI 1.00–1.02, per 1mg/L increase) (Kuwashiro et al., 2013). The CIAFS-1 trial (NCT02282098) is a feasibility study investigating colchicine treatment in patients with AF and its effect on D-dimer and hsCRP levels.

### Inflammation and cryptogenic stroke

Associations between pro-inflammatory markers and cryptogenic stroke have been reported. RANTES, IL-4, IFN-γ, eotaxin and MCP-1 were elevated in a case control study of 162 patients with cryptogenic stroke in Sweden (Holmegaard et al., 2021). A longitudinal study of the pro-inflammatory signature of ischaemic stroke subtypes in 600 cases and 600 controls has reported relatively similar inflammatory marker patterns across stroke subtypes in the acute phase, but that CD5 and IL-12β were specifically elevated in cryptogenic stroke (Stanne et al., 2022). In BISC, higher IL-6 levels were associated with recurrent MACE and stroke in those whose index event was of undetermined cause (IL-6, MACE: RR 1.78, 95% CI 1.19-2.66; IL-6, recurrent stroke: RR 1.65, 95% CI 1.09–2.5; CRP, MACE: RR 1.45, 95%CI 1.04–2.03) (ESOC, 2023).

## Anti-inflammatory treatment for stroke prevention

### Statins

Statins have pleiotropic effects including anti-inflammatory effects in clinical and experimental studies (Sacks et al., 1996). The JUPITER trial randomized patients without established cardiovascular disease, low LDL (<3.4mmol/L) but with raised baseline hsCRP>2mg/L to rosuvastatin 20mg or placebo. Rosuvastatin therapy reduced LDL by 50%, hsCRP by 37%, and almost halved the risk of MACE [HR 0.56 (95% CI 0.46–0.69)]. For the outcome of stroke alone the risk was also halved (HR 0.52 95% CI 0.34–0.79) (Ridker et al., 2008). A meta-analysis of 46 studies in patients with cardiovascular disease reported that statin therapy reduced blood hsCRP levels by −0.97mg/L, (95% CI −1.26 to −0.68) (Kandelouei et al., 2022).

### Specific anti-inflammatory agents

In 2017, publication of CANTOS was a watershed moment as the first randomized trial proving the benefit of specific inhibition of the NLRP3-IL-1β-IL-6-CRP inflammatory cascade to reduce vascular events (Ridker et al., 2017). Over 10,000 patients with prior myocardial infarction and hsCRP ≥2mg/L were randomized to treatment with canakinumab (a monoclonal antibody against IL-1β) in addition to usual care. At 2 years, the canakinumab arm had lower hsCRP levels, and at 3.7 years, the primary outcome (MACE) was reduced by 15% (OR 0.85, 95% CI 0.74–0.98) with greatest benefit in patients with lowest on-treatment hsCRP and IL-6. However, cost and safety considerations (neutropenia and death due to infection in the canakinumab arm) has limited its translation to mainstream practice.

Tocilizumab, a monoclonal antibody which inhibits binding of IL-6 to its receptor, was studied in a small RCT of 200 patients with STEMI and demonstrated modest improvement in early myocardial salvage but no difference in clinical outcomes at 6 months (Broch et al., 2021). Tocilizumab treatment was associated with reduced CRP levels but increased LDL, triglycerides and liver enzymes levels, raising concern about safety in patients with cardiovascular disease. To our knowledge, tocilizumab has not been specifically studied for stroke prevention.

In the CIRT trial, 4786 patients with coronary heart disease and either type 2 diabetes or the metabolic syndrome were randomized to low-dose methotrexate or placebo. After 2.3 years, low-dose methotrexate did not result in lower IL-1β, IL-6 or CRP levels compared to placebo, and there was no difference in the risk of the primary composite outcome of MACE (HR 0.96, 95% CI 0.79–1.16) or for nonfatal stroke alone (HR 0.91, 95% CI 0.54–1.52) (Ridker et al., 2019). Incidence of infection, gastrointestinal disorders, and skin cancers were also higher in the methotrexate treated arm.

The need for a safe, efficacious, tolerable and cost-effective anti-inflammatory agent targeting vascular disease has generated interest in colchicine. Colchicine has been used as a gout treatment for centuries and has established safety and tolerability when used at low doses for prevention of gout, Bechet's and Familial Mediterranean Fever. Colchicine acts by inhibiting microtubule formation, impairing inflammatory cell mitosis, migration, and oxidative stress (Martínez et al., 2018). Other data suggests that colchicine prevents activation of the NLRP3 inflammasome, thus inhibiting activation of IL-1β and secondary pro-inflammatory cytokine release (Martinon et al., 2006). A meta-analysis of randomized controlled trials in 11,816 patients with coronary disease reported a reduction of 25% in myocardial infarction, stroke or cardiovascular death in patients treated with low-dose colchicine, without any difference in all-cause mortality. For the outcome of stroke alone, colchicine therapy reduced the risk by 46% (RR 0.54, 95% CI 0.34–0.96) (Fiolet et al., 2021). There were more gastrointestinal adverse events in colchicine-treated patients, but these were generally mild. No major safety concerns were identified. Colchicine at a dose of 0.5 mg has been introduced in the European Society of Cardiology guidelines for secondary prevention in patients with coronary artery disease, to be considered if recurrent cardiovascular risk is very high despite maximum (tolerated) conventional treatments (Tardif et al., 2019; Nidorf et al., 2020; Visseren et al., 2021).

The ongoing CONVINCE trial (COlchicine for preventioN of Vascular Inflammation in Non-CardioEmbolic stroke) seeks to establish whether addition of low-dose colchicine to usual care after ischaemic stroke will result in reduced recurrent stroke and cardiovascular events (Kelly et al., 2021a). CONVINCE includes patients with large artery, small vessel strokes, or strokes of undetermined cause, excluding those with cardioembolism or other determined cause. With a sample size of 3,154, it is powered to detect a 25% risk reduction after a median of 36 months follow up and is expected to report in 2024.

In China, the recently completed CHANCE-3, examined the effect of acute (<24 h) colchicine therapy in patients with high-risk minor stroke and TIA and hsCRP ≥ 2 mg/L for prevention of all stroke at 90 days, but reported no difference in the primary outcome (HR 0.98, 95% CI 0.83–1.16) (Wang et al., 2023). CASPER (ACTRN12621001408875) in Australia will test colchicine for reduction of recurrent stroke and MACE in patients with persistently raised hsCRP≥2mg/L after index ischaemic stroke. CoVasc-ICH (NCT 05159219) is a feasibility study randomizing patients after intracranial hemorrhage to colchicine and evaluating feasibility, recruitment rate and medication adherence. Other trials focusing on anti-inflammatory agents in stroke disease are outlined in Table 1.

**Table 1:** Randomized controlled trials of anti-inflammatory agents for prevention of recurrent events after stroke and CAD.

**Trial location**	**Registration ID**	**Design**	**Population**	**Intervention**	**Primary outcome, follow-up time**	**Status**
**Colchicine: targeting NLRP3 inflammasome**						
CONVINCE, Europe, Canada	NCT02898610	RCT Open label, blinded endpoint assessed3154 participants	Non severe IS (mRS ≤ 3) or high risk TIA (ABCD2 ≥ 4) Excluding CE	Colchicine 0.5mg OD vs. Usual care	MACE *^**+**^*36 months follow up	Recruitment completed expected results 2024 (Kelly et al., 2021b)
Chance 3, China	NCT05439356	RCTDouble-blindPlacebo controlled 8238 participants	Minor IS or TIAhsCRP ≥ 2mg/L	Colchicine loading then 0.5mg Vs Placebo	**Stroke** (ischaemic or haemorrhagic) 90 days follow-up	Reported at WSC 2023: no difference in primary outcome, HR 0.98 (0.83–1.16) (Wang et al., 2023)
Casper Australia	ACTRN12621001408875	RCT Not yet recruiting	Minor IS and hsCRP ≥ 2mg/L^*^	Colchicine 0.5mg OD	MACE *^**+**^*biomarker, MRI and PET substudies planned	Not yet recruiting
CO-VASC-ICH Canada	NCT05159219	RCTFeasibility100 participants	Spontaneous ICH < 48 h. History of atherosclerosisor ≥ 2 vascular risk factors	Colchicine 0.5 mg OD VsPlacebo	Recruitment rate retention rate medication adherence	Recruiting
RIISC-THETIS France	NCT 05476991	Factorial RCT 2x2Open label 2800 participants	Imaging proven infarct + Evidence of carotid atherosclerosis	Colchicine 0.5mg Vs no colchicine and Ticagrelor 90mg BD Vs Aspirin	MACE*^**+**^*36 months follow up	Start 2023 estimated completion 2027
CIAFS-1 Canada	NCT02282098	RCT feasibility	AF on OAC x3/12	Colchicine 0.6 mg BD vs. placebo	D-dimerhs CRP 3 months	Completed 2021 awaiting results
**Targeting IL-1**β						
CANTOS; Ridker et al. (2017)	Ridker et al., NEJM 2017	RCTDouble blind 10,061 participants	Prior MI hs CRP ≥ 2 mg/L	Canakinumab 50 mg vs 150 mg vs 300 mg SC vs. placebo	MACE*^**+**^* 48 months	50mg: HR 0.93 (0.80–1.70)150 mg: HR 0.85 (0.74–0.98) 300 mg: 0.86 (0.75–0.99) higher fatal infections no difference all-cause mortality
**Targeting IL-6**						
RESCUE; Ridker et al. (2021)	Ridker et al., Lancet 2021	RCT 264 participants phase 2	CKD hsCRP ≥ 2mg/L	Ziltivekimab SC monthly7.5 mg vs. 15 mg vs. 30 mg vs. placebo	Change in hsCRP 24 weeks	7.5mg: ↓77% 15 mg: ↓88% 30 mg: ↓90% placebo: ↓4% ← → cholesterol no excess SAEs
ZEUS USA	NCT05021835	RCT6200 participants	CKD eGFR ≥ 15–60 ml/min/1.73m2hsCRP ≥ 2 mg/L evidence of atherosclerosis	Ziltivekimab 15 mg vs. Ziltivekimab 30 mg SC monthly vs. placebo SC injection	Time to MACE*^**+**^* 48 months	Started 2021 estimated completion 2025
**Other agents**						
Methotrexate CIRT; Ridker et al. (2019)	Ridker et al., NEJM 2019	RCT4786 participants	Previous MI or CAD T2DM or metabolic syndrome	MTX 15-20mg weekly Vs placebo	MACE*^**+**^* 2.3 years follow up IL-1β, IL-6 and CRP levels	No difference HR 0.96 (0.79–1.16) higher levels of adverse events MTX.

IS, ischaemic stroke; TIA, transient ischaemic attack; RCT, randomized controlled trial; CE, cardioembolism; MI, myocardial infarction; MTX, methotrexate; CAD, coronary artery disease; AF, atrial fibrillation; T2DM, type 2 diabetes mellitus; SAE, serious adverse events; SC, subcutaneous injection; IV, intravenous injection; NIHSS, National Institute of Health Stroke Scale; mRS, modified Rankin scale; eGFR, estimated Glomerular filtration rate; hsCRP, high sensitivity C-reactive protein; NFκB, nuclear factor kappa-B; ICH, intracerebral hemorrhage.

* hsCRP measured 4-52 weeks after acute stroke event.

+ MACE definitions.

CONVINCE, nonfatal ischaemic stroke, nonfatal major cardiac event (unstable angina, myocardial infarction or cardiac arrest) or vascular death.

CANTOS, ZEUS, nonfatal myocardial infarction, nonfatal stroke, cardiovascular death.

RIISC THETIS, nonfatal ischaemic stroke, stroke of any cause, myocardial infarction, coronary or carotid revascularisation or vascular death.

CIRT, nonfatal myocardial infarction, nonfatal stroke, hospitalization for unstable angina leading to urgent revascularisation, cardiovascular death.

### Future steps and controversies

Multiple drug candidates exist which target the NLRP3 inflammasome to IL-1β to IL-6 to CRP inflammatory pathway, but one of the most promising is ziltivekimab, a human monoclonal antibody against IL-6 ligand. This drug is currently being tested in patients with chronic kidney disease (CKD stages 3–5) for whom vascular risk is high and colchicine (which is renally excreted) is contraindicated. A phase II randomized trial, in patients with chronic kidney disease, CRP >2mg/L and NTproBNP >250 pg/mL, has demonstrated that 24 weeks of treatment with ziltivekimab substantially reduced serum levels of hsCRP as well as fibrinogen, serum amyloid A, haptoglobin, phospholipase A2 and lipoprotein(a) without major safety concerns (Ridker et al., 2021). It is as yet unknown if this will translate to reduction in clinical vascular events. The ongoing randomized controlled trial ZEUS (NCT05021835) aims to evaluate this question in 6,200 patients with CKD, CRP > 2 mg/L and atherosclerotic cardiovascular disease, including symptomatic carotid stenosis.

Another promising approach for clinical benefit is the potential synergy of intensive lipid-lowering and anti-inflammatory combination therapy. Proprotein convertase subtilisin-kexin type 9 inhibitors (PCSK9) are proven to substantially reduce LDL levels and recurrent events in patients after acute coronary syndrome and stroke, whose LDL is not supressed to target with high intensity statin therapy (Sabatine et al., 2017; Schwartz et al., 2018). Two PCSK9 trials (FOURIER and SPIRE 1 and 2) demonstrated that PCSK9 inhibition in addition to statin treatment reduces LDL levels but was not associated with significant reduction in hsCRP. However, rates of cardiovascular events were higher for patients with higher residual hsCRP levels compared with lower despite intensive lipid-lowering, supporting the concept of residual inflammatory risk (Pradhan et al., 2018). In FOURIER, PCSK9 inhibition with evolocumab reduced recurrent vascular events across all hsCRP strata, with greater absolute reduction in patients with higher baseline hsCRP (Bohula et al., 2018). Additionally, PCSK9 inhibition has been associated with carotid plaque lipid core regression, in a small observational MRI study (Lepor et al., 2021). In stroke, an attractive target for future study may be a factorial trial investigating the combination of anti-inflammatory therapy with LDL lowering therapy based on baseline LDL/hsCRP profiles and objective evidence of atherosclerosis.

## Author contributions

SG drafted the article. JM and PK revised and contributed content. All authors approved the final manuscript.
